# The effect of umbilical cord cleansing with chlorhexidine on omphalitis and neonatal mortality in community settings in developing countries: a meta-analysis

**DOI:** 10.1186/1471-2458-13-S3-S15

**Published:** 2013-09-17

**Authors:** Aamer Imdad, Luke C Mullany, Abdullah H Baqui, Shams El Arifeen, James M Tielsch, Subarna K Khatry, Rasheduzzaman Shah, Simon Cousens, Robert E Black, Zulfiqar A Bhutta

**Affiliations:** 1 Division of Women and Child Health, Aga Khan University, Karachi, Pakistan; 2 Department of International Health, Johns Hopkins Bloomberg School of Public Health, Baltimore, USA; 3 International Centre for Diarrheal Diseases Research, Bangladesh; 4 Nepal Nutrition Intervention Project-Sarlahi, Kathmandu, Nepal; 5 London School of Tropical Medicine & Hygiene, London, UK

## Abstract

**Background:**

There is an increased risk of serious neonatal infection arising through exposure of the umbilical cord to invasive pathogen in home and facility births where hygienic practices are difficult to achieve. The World Health Organization currently recommends ‘dry cord care’ because of insufficient data in favor of or against topical application of an antiseptic. The primary objective of this meta-analysis is to evaluate the effects of application of chlorhexidine (CHX) to the umbilical cord to children born in low income countries on cord infection (omphalitis) and neonatal mortality. Standardized guidelines of Child Health Epidemiology Reference Group (CHERG) were followed to generate estimates of effectiveness of topical chlorhexidine application to umbilical cord for prevention of sepsis specific mortality, for inclusion in the Lives Saved Tool (LiST).

**Methods:**

Systematic review and meta-analysis. Data sources included Cochrane Central Register of Controlled Trials (CENTRAL) in the Cochrane Library, PubMed, CINHAL and WHO international clinical trials registry. Only randomized trials were included. Studies of children in hospital settings were excluded. The comparison group received no application to the umbilical cord (dry cord care), no intervention, or a non-CHX intervention. Primary outcomes were omphalitis and all-cause neonatal mortality.

**Results:**

There were three cluster-randomised community trials (total participants 54,624) conducted in Nepal, Bangladesh and Pakistan that assessed impact of CHX application to the newborn umbilical cord for prevention of cord infection and mortality. Application of any CHX to the umbilical cord of the newborn led to a 23% reduction in all-cause neonatal mortality in the intervention group compared to control [RR 0.77, 95 % CI 0.63, 0.94; random effects model, I^2^=50 %]. The reduction in omphalitis ranged from 27 % to 56 % compared to control group depending on severity of infection. Based on CHERG rules, effect size for all-cause mortality was used for inclusion to LiST model as a proxy for sepsis specific mortality.

**Conclusions:**

Application of CHX to newborn umbilical cord can significantly reduce incidence of umbilical cord infection and all-cause mortality among home births in community settings. This inexpensive and simple intervention can save a significant number of newborn lives in developing countries.

## Introduction

Since 1998, the World Health Organization has recommended promotion of clean and dry cord care for newborn infants, while noting that topical antiseptics may be used where risk of infections is high [1]. A Cochrane review by Zupan et al included 21 trials involving 8959 subjects and covered all types of antiseptics applied to umbilical cord [2]. All of the included studies were conducted in hospital settings and, with the exception of one trial from Thailand, in high income countries. There were no systemic infections or deaths, the primary outcomes considered by the review, in any of the trials. Risk of umbilical cord infection was approximately half as likely when a topical antiseptic was applied, as compared with dry cord care or placebo, but the combined result did not reach statistical significance (RR = 0.53; 95% CI 0.35 –1.13). Topical triple dye seemed to be more effective than alcohol (RR= 0.30; 95% CI: 0.19–0.49) or povidone-iodine (RR = 0.15, 95% CI: 0.07–0.32) in preventing cord infection, however no specific recommendations were made in favor or against of an antiseptic, as the overall evidence was inconclusive.

While umbilical cord infections can occur in all settings, they are more likely to occur in low-income countries, where the majority of births take place at home in unclean settings and are not attended by a skilled attendant [3]. Since all but one of the aforementioned trials were conducted in hospitals in high income countries, the results cannot be generalized to community settings in low income countries where achieving clean and dry cord care is difficult in general [4,5].

Recently, three large community-based randomized trials have been conducted in Nepal, Bangladesh and Pakistan to study the effectiveness of application of 4.0% chlorhexidine (CHX) to the umbilical cord after birth. The objective of this study was to undertake a meta-analysis of these studies to evaluate the effect of application of chlorhexidine to the umbilical cord for prevention of omphalitis and neonatal mortality in community settings. This paper is a part of series of papers for Lives Saved Tool (LiST) model which is a computer-based model that estimates the impact of increasing coverage of different intervention packages and coverage levels for individual countries, states or districts [6]. The ultimate goal of LiST tool is to provide a structured format for program managers or ministry of health personnel to combine the best scientific information about effectiveness of interventions for maternal, neonatal and child health with information about cause of death and current coverage of interventions to inform their planning and decision-making, to help prioritize investments and evaluate existing programs. An intervention is currently included in the LiST if there is substantial evidence that it decreases maternal mortality, neonatal/child mortality and/or stillbirths. The process of inclusion of a particular intervention to LiST tool is guided by qualitative assessment of available evidence according to Grading of Recommendations, Assessment, Development and Evaluation (GRADE) criteria and quantitative inputs according to Child Health Epidemiology Reference Group (CHERG) guidelines [6,7].

## Methods

### Literature search

We searched the Cochrane Central Register of Controlled Trials, PubMed and CINHAL. All searches were conducted on November 22, 2012. We used the following search strategy on PubMed: ("Chlorhexidine"[Mesh] OR Chlorhexidine* AND "Umbilical Cord"[Mesh]) OR "Umbilicus"[Mesh]). No limits were applied to this search strategy. To identify ongoing and unpublished trials, we used the WHO international clinical trials registry, which searches multiple trial registries. Reference lists of reviews, included studies, and excluded studies were also searched for additional citations. We contacted organizations and researchers by email and by phone to obtain additional data if required.

### Eligibility criteria

#### Types of trials

Community randomized controlled trials including cluster trials and factorial trials were included irrespective of publication status or language.

#### Types of participants

Neonates born alive to women enrolled in the study. Studies conducted entirely in hospital settings were excluded.

#### Types of interventions

Included studies examined effectiveness of topical application of 4.0 % chlorhexidine to newborn umbilical cord for prevention of omphalitis and neonatal mortality. The comparison group received dry cord care, no intervention or non CHX intervention.

### Types of outcome measures

The primary outcomes were incidence of sepsis-specific mortality, all-cause mortality and omphalitis in the neonatal period. Neonatal period was defined as first 28 days of life. We also examined cord separation time in the intervention group compared to control. For the outcome omphalitis, three case definitions were used:

• Algorithm 1: Moderate or severe redness

• Algorithm 2: Moderate redness with pus, or severe redness (without regard to pus)

• Algorithm 3: Severe redness with pus

### Data abstraction and risk of bias assessment

All the included trials were assessed for methodological quality and outcomes of interest using a standardized form [7]. Two reviewers (AI and ZAB) independently assessed the studies for inclusion. Data were double abstracted. Data were abstracted for study design, study site, study methods and outcomes of interest. Risk of bias in the included studies was assessed with the Cochrane Collaboration’s risk of bias tool [8]. This assessment is based on five criteria that includes bias from sequence generation, allocation concealment, blinding of participants, assessors, and providers, selective outcome reporting and incomplete data. Risk of bias for each domain is rated as high (seriously weakens confidence in the results), low (unlikely to seriously alter the results), or unclear.

Individual studies were graded according to CHERG adaptation of GRADE technique. In this method of qualitative evaluation, all randomized trial received an initial score of ‘high’ and an observational study as ‘low’. The study scores were adjusted depending on limitations of the study design. Trials with a final grade of ‘high’ or ‘moderate’ and ‘low grade’ were included in the analysis with exclusion of studies with a final grade of ‘very low’ [7].

### Quantitative data synthesis

Meta-analyses were conducted where data were available from more than one study for an outcome. The results were presented as risk ratios (RR) and 95% confidence intervals (CIs). Random effects meta-analysis was used for all analyses. For cluster randomized trials, we used the reported cluster-adjusted risk ratio and 95% confidence interval, irrespective of the method used [9]. The value of design effect was taken as stated in the study or was inferred from intra-cluster correlation coefficient. Pooled estimates of the evaluated outcome measures were calculated by the generic inverse variance method. This method is a common and simple version of the meta-analysis procedure and is so named because the weight given to each study is chosen to be the inverse of the variance of the effect estimate (i.e. one over the square of its standard error) [9]. In this way larger studies, which have smaller standard errors, are given more weight than smaller studies, which have larger standard errors. This minimizes the imprecision (uncertainty) of the pooled effect estimate. The assessment of statistical heterogeneity among trials was done by visual inspection i.e. the overlap of the confidence intervals among the studies, Chi square (P-value) of heterogeneity in the meta-analyses and I^2^ value. A low P value (less than 0.10) or a large I^2^ statistic (I^2^ >50 %) was considered as evidence of significant heterogeneity, which was explored further by sensitivity analysis as required. All meta-analyses were conducted using software Review Manager version 5.1 [10]. Qualitative assessment of pooled data was presented in “Summary of findings” table according to the Child Health Epidemiology Group (CHERG) adaptation of the GRADE criteria [7].

The three included studies comprised 10 study groups. In order to obtain a summary effect estimate from all the CHX groups, we combined the groups as shown in table 1. This approach was adopted based on expert opinion canvassed at a meeting held at John Hopkins University in January 2010 and further sensitivity analysis was undertaken to explore possible bias.

### Recommendations for LiST

We followed standardized guidelines of Child Health Epidemiology Reference Group to get a point estimate for effectiveness of application of chlorhexidine to the umbilical cord for prevention of sepsis-specific neonatal mortality in community settings. These rules were applied to collective mortality and morbidity outcomes to get a most appropriate estimate for inclusion in the LiST model. The final decision about the best estimate is based on three components 1) the volume and consistency of the evidence 2) the size of risk ratio and 3) the strength of the statistical evidence for an association between the intervention and outcome, as reflected by the p-value. More details about application of CHERG rules are provided in the methods paper [7].

## Results

### Trial flow

**Figure 1:**
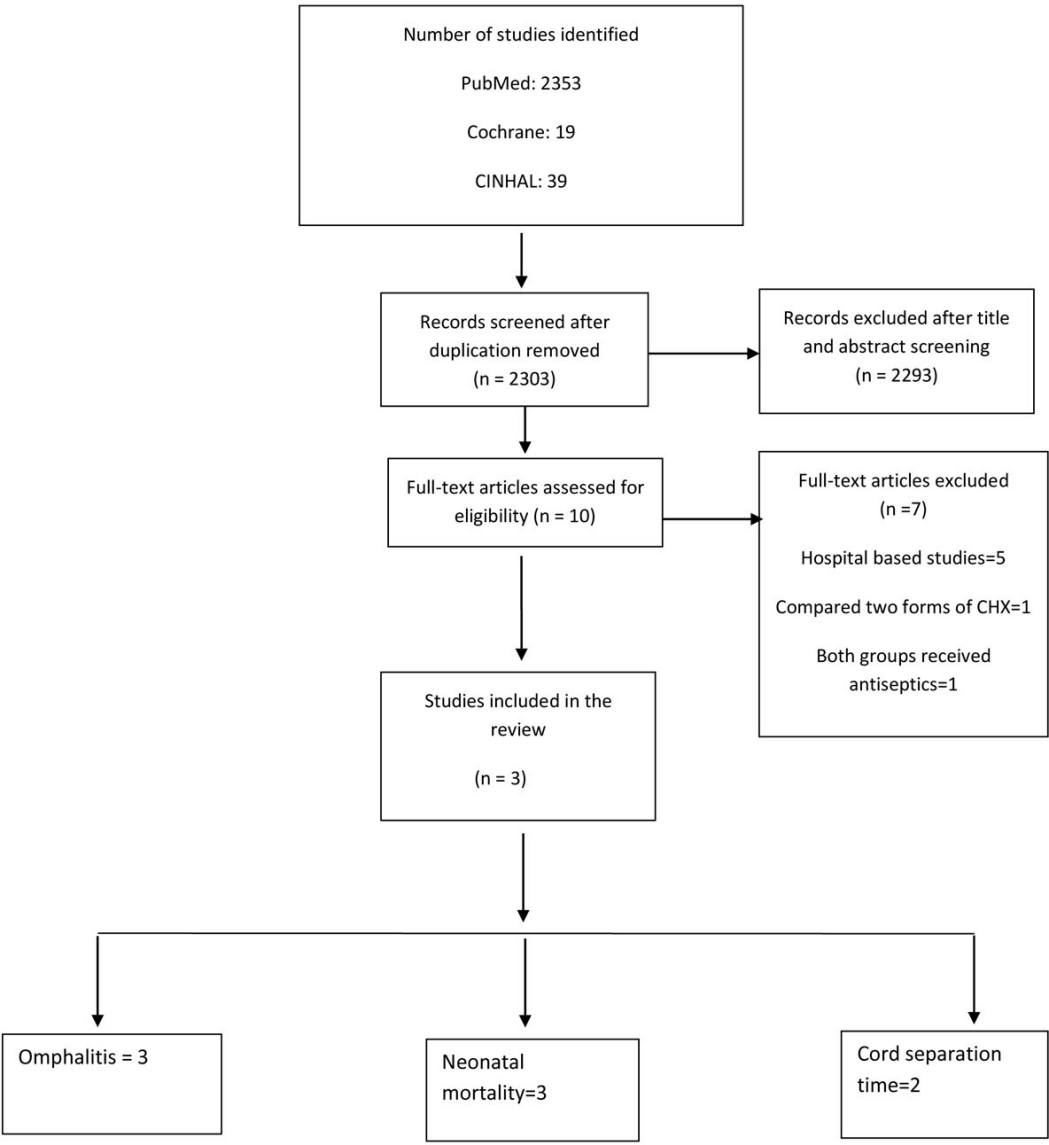
Flow diagram showing identification of studies

The literature search of electronic databases, and papers from hand searches yielded a total of 2303 titles after removal of duplicates (Fig 1). After initial screening of titles and abstracts, 10 studies were selected for data abstraction. Of these 7 were excluded because they were hospital-based studies (n=5), or compared two different forms of CHX (n=1) or both groups had received antiseptics (n=1). The three included trials were conducted in Nepal, Bangladesh, and Pakistan [11-13]. A search of WHO’s international clinical trials registry and communication with investigators in the field revealed two ongoing community-based randomized trials, one in Tanzania [14] and one in Zambia [15].

### Study characteristics

Table 2 describes the characteristics of included studies. All three included studies from Nepal, Bangladesh and Pakistan were cluster randomized community trials. The total number of participants in the three trials was 54,624. Duration of follow up in all the studies was the first 28 days of life except in the Pakistan trial where a 6 month follow up visit was also made. The overall duration of CHX application in CHX intervention groups (A & C) were 11.1 ± 2.8 days with a mean 2.4 ± 0.7 applications per/day in Pakistan trial [13]. In Bangladesh and Nepal trials, all babies were enrolled if alive at the first home visit by project staff, and the first visit took place within 7 (Bangladesh) and 10 (Nepal) days after birth. In Pakistan, babies who were live born and delivered by participating TBAs were enrolled. In the Bangladesh trial, high intervention coverage was achieved for enrolled newborns and less than 1% of the participants received no intervention visit [12]. Both the Nepal and Bangladesh trials included hospital born babies. The average number of home visits per newborn was 6.5 out of 7 and 84.0% of the enrolled newborns received the complete 7 days of intervention [12]. In the Pakistan trial, about 97% of enrolled infants received at least one CHX application. In the Nepal trial, effective coverage was about 96.3 % and the average number of home visits was 5.9 ± 1.5 days out of 7 days [11]. Additional file 1 summarises the risk of bias in included studies according to latest Cochrane handbook [8].

**Table 1:** Scheme of analysis for chlorhexidine groups and non-chlorhexidine groups in the included studies

Study ID	Study groups	Any CHX vs. No CHX
**Nepal trial 2006 **[11]	**Group A**: Multiple cleansing of cord stump with 4 % CHX. **Group B:** Cleansing of cord with Soap and water **Group C:** Dry cord care	**Group A vs. Group B +C**

**Bangladesh trial 2012 **[12]	**Group A:** Multiple cleansing of cord stump with 4 % CHX. **Group B:** Single cleansing of cord stump with 4 % CHX. **Group C:** Dry cord care	**Group A + B vs. Group C**

**Pakistan trial 2012 **[13]	**Group A:** Multiple cleansing of cord stump with 4 % CHX and promotion of handwashing among caregivers. **Group B:** Promotion of handwashing only. **Group C:** Multiple cleansing of cord stump with 4 % CHX only **Group D:** Dry cord care	**Group A +C vs. Group B +D**

CHX: Chlorhexidine

**Table 2:** Characteristics of included studies

Study characteristics	Nepal trial*	Bangladesh trial	Pakistan trial
**Type of study**	Cluster RCT	Cluster RCT	2 × 2 factorial design cluster RCT
**No. clusters (average size)**	413 (700)	133 (4100)	187 (1000)
**Sample size total**	15,123	29,760	9,741
**Average sample size per group**	~ 5,050	~ 9,900	~ 4,850
**Duration of trial**	Nov 2002 to Mar 2005	Jun 2007 to Sep 2009	Jan 2008 to Jun 2009
**Overall NMR**	32/1000	36/1000	30/1000
**% of home births**	92 %	93 %	80 %
**Inclusion criteria**	All live births in the study area	All live births in the study area	All live births in the study area were included except those who were born in hospitals.
**Exclusion criteria**	Not met within 10 days after birth	Didn’t receive intervention within 7 days after birth	Not met within 3 days after birth. Babies with congenital anomalies
**Comparison group**	Dry cord care	Dry cord care	Dry cord care
**Intervention groups**	1.Multiple CHX 2.Soap/H_2_O	1.Multiple CHX 2.Single CHX	1.Multiple CHX 2.Handwashing (HW) 3.CHX + HW
**CHX concentration**	4.0 %	4.0 %	4.0 %
**Participants recruiters**	Local female worker	CHWs	CHWs
**Intervention providers**	Local project staff	Village health worker	TBA to caretaker
**Outcome assessors**	(Nonmedical) field workers	CHWs	CHWs
**Basic interventions to all babies/mothers**	CDK, FE/FA, TT, promotion of ANC/ENC	CDK. FE/FA, promotion of TT, ANC, birth preparedness, ENC	Basic component of ENC as promoted by Ministry of Health
**Primary outcomes**	Omphalitis, neonatal mortality	Omphalitis, neonatal mortality	Omphalitis, neonatal mortality
**Follow up days**	1, 2, 3, 4, 6, 8, 10, 12, 14, 21, 28	1,3,6,9,15,28	1,3,5,7,14,28

RCT: Randomized Controlled trial, CHX: chlorhexidine, CDK: clean delivery kit, Fe/FA: Iron/Folic acid, TT, Tetanus oxide, ANC: Antenatal care, ENC: essential newborn care, CHW: Community Health Workers, TBA: Traditional Birth Attendant

* Nepal trial was nested within a study of the effect of full-body skin cleansing with antiseptic on neonatal mortality. In that trial, newborns were given a single fullbody wipe with either 0·25% chlorhexidine or placebo solution immediately after birth. In each skin cleansing group (0·25% chlorhexidine or placebo) in the main trial, sectors were randomized to one of three cord-care regimens (Which makes total of 6 study groups).

### Quantitative data synthesis

#### Effect on sepsis specific mortality

The Nepal trial reported data on sepsis-specific mortality and reported a 31 % reduction in CHX group compared to dry cord care however the results were not statistically significant (RR=0.69, 95 % CI 0.40-1.18) [11]. The overall quality grade for this outcome was that of “low” level (Table 3).

**Table 3:** Summary of findings of trials to assess the effect of application of CHX to newborn’s umbilical cord

Quality Assessment						Summary of findings		
				Generalizability		Number of cases		Pooled effect

No. of studies	Design	Limitations	Consistency	Generalizability to Population of Interest	Generalizability to intervention of Interest	CHX	Control	Relative risk (95 % CI)

Sepsis Specific mortality: GRADE quality: Low								

1	RCT	None	Only one trial reported data	All the participants were neonates	4.0% chlorhexidine solution.	Only RR was reported in the published manuscript.		0.69 (0.40-1.18)

All-Cause neonatal mortality: GRADE quality: Moderate								

3	RCT	All three included studies are well conducted community randomized trials. Intervention was not masked in two studies.	Direction of effect in favor of intervention in all three studies. There was moderate statistical heterogeneity (I^2^ =50%).	All the participants were neonates	All the studies used 4.0% chlorhexidine solution. Frequency of application was different in different study groups.	670/29543	655/25072	0.77 (0.63-0.94)

Incidence of omphalitis: Algorithm 1 GRADE quality: Moderate								

3	RCT	All three included studies are well conducted community randomized trials. Intervention was not masked in two studies.	Direction of effect in favor of intervention in all three studies. There was moderate statistical heterogeneity (I^2^ =34%).	All the participants were neonates	All the studies used 4.0% chlorhexidine solution. Frequency of application was different in different study groups.	Not applicable as data was pooled by generic inverse variance	Not applicable as data was pooled by generic inverse variance	0.73 (0.64-00.83)

Incidence of omphalitis: Algorithm 2 GRADE quality: High								

3	RCT	All three included studies are well conducted community randomized trials. Intervention was not masked in two studies.	Direction of effect in favor of intervention in all three studies. No statistical heterogeneity (I^2^ =0%).	All the participants were neonates	All the studies used 4.0% chlorhexidine solution. Frequency of application was different in different study groups.	Not applicable as data was pooled by generic inverse variance	Not applicable as data was pooled by generic inverse variance	0.69 (0.60-0.79)

Incidence of omphalitis: Algorithm 3 GRADE quality: High								

3	RCT	All three included studies are well conducted community randomized trials. Intervention was not masked in two studies.	Direction of effect in favor of intervention in all three studies. Small statistical heterogeneity (I^2^ =19 %).	All the participants were neonates	All the studies used 4.0% chlorhexidine solution. Frequency of application was different in different study groups.	Not applicable as data was pooled by generic inverse variance	Not applicable as data was pooled by generic inverse variance	0.46 (0.32-0.66)

• The GRADE assessment is based on

1) The volume and consistency of the evidence.

2) The size of summary estimate and

3) The strength of the statistical evidence for an association between the intervention and outcome.

• The quality grade can be interpreted as follows:

High quality— Further research is very unlikely to change our confidence in the estimate of effect

Moderate quality— Further research is likely to have an important impact on our confidence in the estimate of effect and may change the estimate

Low quality— Further research is very likely to have an important impact on our confidence in the estimate of effect and is likely to change the estimate

Very low quality— Any estimate of effect is very uncertain

• This GRADE table is an adaptation form CHERG methods paper[7] and GRADE paper[26]

#### Effect on all-cause mortality

**Figure 2:**
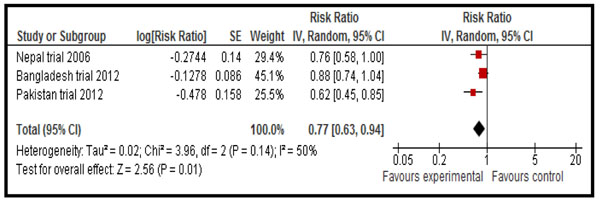
Effect of chlorhexidine cord cleansing on all-cause neonatal mortality

There were 1325 deaths in all study groups and the combined results for “any CHX vs. No CHX” showed an important reduction of 23% in all-cause neonatal mortality (RR = 0.77, 95 % CI 0.63 - 0.94; random model, I^2^=50 %] (Fig 2). The quality of the evidence was graded as ‘moderate’ for this outcome (Table 3). When restricting analysis to facility births only (available from Nepal and Bangladesh) there was no indication that the impact of chlorhexidine was lessened (RR=0.50 [0.27 - 0.92]).

#### Omphalitis

**Figure 3:**
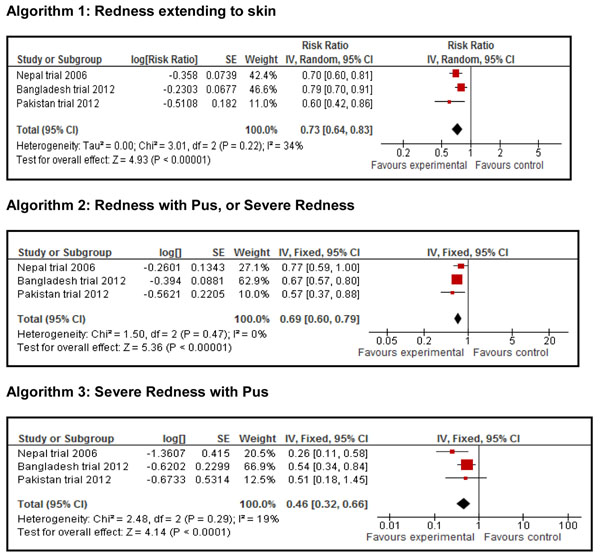
Effect of chlorhexidine application to newborn’s umbilical cord. Any *Chlorhexidine vs. No Chlorhexidine*: Incidence of Omphalitis according to severity. Algorithm 1: Redness extending to skin. Algorithm 2: Redness with Pus, or Severe Redness. Algorithm 3: Severe Redness with Pus

Data for omphalitis were analyzed according to three different algorithms used to define omphalitis. Figure 3 shows the meta analytic results. CHX was associated with reductions in omphalitis using all three algorithms with the largest reduction observed for severe omphalitis (RR = 0.46, 95 % CI 0.32-0.66). The quality of the evidence was graded as ‘moderate” for mild omphalitis and ‘high’ that for moderate and severe omphalitis.

#### Cord separation time

In the Nepal trial, cord separation time was longer in the CHX group (5.32 ± 2.4 days) compared to the dry cord care group (4.24 ± 1.6 days). In the Pakistan trial, there was no difference in the time to separation of the cord between each group [CHX group 6.2 ± 1.3 days, group HW 5.9 ± 1.5, groups CHX + HW and dry cord care both 6.0 ± 1.6 days]*.* In Bangladesh, separation time in the combined chlorhexidine group (7.20 ± 3.0 days) was 2.40 (95% CI: 2.17 – 2.64) days longer than among babies not exposed to chlorhexidine (4.79 ± 1.8 days).

#### Application of CHERG rules

We followed standardized guidelines of Child Health Epidemiology Reference Group to get a point estimate for effectiveness of chlorhexidine application of chlorhexidine to the umbilical cord for prevention of sepsis-specific neonatal mortality in community settings. Table 4 gives the details of application of CHERG rules. Estimate for all-cause mortality was recommended to be included to LiST model.

**Table 4:** Application of CHERG Rules for selection of point estimate for sepsis mortality for inclusion to LiST model [7]

Outcome measure	Studies	Total Events	Effect size	GRADE quality of pooled estimate	Application of standard rules
Sepsis specific neonatal mortality	(n=1)	Not reported	31 % reduction, statistically non-significant (RR=0.69, 95 % CI 0.40-1.18)	Low	Effect size for sepsis specific mortality was not used for inclusion in LiST model as results were not statistically significant and overall quality grade was “low”

All cause neonatal mortality	(n=3)	838	23% reduction, Statistically significant (RR=0.77, 95 % CI 0.63 - 0.94)	Moderate → Low	Effect size for all-cause mortality was used for inclusion to LiST model. This was based on rule 1 that says that if there is no evidence for cause specific mortality and there is evidence for all-cause mortality, use the effect size for all-cause and down grade quality grade by one.

## Discussion

The results of this meta-analysis indicate that application of CHX to the newborn umbilical cord substantially reduced all-cause neonatal mortality and omphalitis in 3 low resource settings in Asia. All-cause mortality among newborns was reduced by 23% in the CHX group compared to controls. The reduction in incidence of omphalitis ranged from 27% to 54% depending on the severity of infection.

### Strengths and limitation

This meta-analysis evaluates the effect of umbilical cord cleansing by CHX aqueous solution on neonatal mortality and omphalitis in community settings. Another recently published review of chlorhexidine trial reported similar findings [16]. For the primary outcome, neonatal mortality, the evidence is striking. Three studies comprising 10 study groups involving 54,624 participants were analyzed. All the studies reported a protective effect in favor of umbilical cord cleansing by CHX to prevent neonatal death. The quality of the evidence was graded as “moderate” on the GRADE scale (table 3). All studies were randomised with appropriate methods for sequence generation and the study and control groups were comparable in all studies. It was not feasible to mask the intervention for participants and providers, so masking was not done in two studies. Failure to mask and missing data are unlikely to have biased the results as attrition was balanced. Risks of selective outcome reporting were low as protocols of studies were available for evaluation of reporting of *a priori* outcomes. An important observation is that all the babies born in the study period of three studies were not included in the analysis as they could not be enrolled. For example in Bangladesh trial there were 770 deaths among enrolled children but 397 children died before they were enrolled. So the effect estimate is not the effect on all neonatal deaths during the study period. In case all the neonatal deaths were accounted in the study period, effect size might have become smaller. This analysis however requires the assumption that the CHX would have no effect on babies that did not receive intervention, which may not be true. Another important limitation of the current analysis is that we did not have segregated data for low birth weight and premature babies. It is well known that risk of sepsis is greater in preterm and low birth weight babies [17] and their prevalence may differ in different parts of the world. It is therefore not well established that if CHX had any differential effect for prevention of mortality in low birth weight or preterm babies.

Study populations in Nepal, Bangladesh and Pakistan were representative of much of the population in Southeast Asia. This implies that the intervention can at least be applied to Southeast Asian region that contribute a significant number of neonatal deaths around the globe [18]. There are no data available from Africa yet however two randomized trial are being conducted there that will further strengthen the evidence in favor or against of the intervention. No immediate side effects were reported in any of the studies. One important consideration is the reported increase in cord separation time. In the Nepal and Bangladesh studies, participants in CHX group had longer cord separation time compared to the controls [19]. In both studies however, there was no additional risk of infection associated with increases in cord separation time. There was no difference in cord separation time in Pakistan trial [13].

### Choice of comparisons

The three included studies had 10 study groups that tested different frequency and duration of CHX application. The comparison groups included dry cord care [11-13], washing of cord with soap/water [11] and promotion of handwashing practices of caregivers [13]. In order to examine whether CHX has any protective effect compared to these comparison groups, all the CHX groups were combined and all the other groups compared in an analysis “any CHX vs. no CHX” as shown in table 1. This combination of study groups are not expected to bias the results as the soap/water group in Nepal study had an effect size very similar to control (i.e. dry cord care). The Pakistan trial was a factorial design trial and we included the factorial analyses in which handwashing groups were balanced between the two study groups (CHX + HW and CHX only versus HW only and Dry cord care). The Bangladesh trial had two CHX groups i.e. 1 day or 7 day application. These were combined to include CHX groups in one arm and compared it with all non-CHX interventions across three studies.

### Choice of model

We used random effects meta-analysis for all analyses. There are no comprehensive rules on when to use random effects or fixed effects models for meta-analysis [8]. The difference between two models is that a fixed effects model assumes that observed differences between results of trials is due to sampling variation of individual studies only whereas a random effects model assumes that outcomes of trials might differ both because of sampling variation of individual studies and true diversity in effects. Both models can be appropriately applied for pooling data but a random effects model is usually preferred with heterogeneity. We used random effects models because there was substantial heterogeneity across studies in study design, settings, and package of interventions and/or intensity of delivery of those interventions. Another reason to use random model was that we assumed that the true effect of CHX does vary across the community settings based on above factors.

### Predictors of statistical heterogeneity

For all-cause mortality, there was moderate statistical heterogeneity (I^2^=50%). One of the likely causes of this heterogeneity was the difference in mortality rates among enrolled babies in the control populations. The mortality in the control group was 36.1/1000 in Pakistan trial, 28.3/1000 in Bangladesh trial and 19.3/1000 in Nepal trial. Other important factors to consider are the home practices for cord care in different communities. For example, in the Pakistan trial, a significant proportion of study population applied lead (called surma in the local language) to the umbilical cord stump, which may cause irritation at the site and provide a port of entry for infectious agents. Other practices across the studies included application of materials such as ash, mud and even cow dung; however their prevalence was very low. It is important to note that these home practices may increase the risk of cord infections but do not explain the overall increased rate of high cord infections in these communities. Furthermore, as the most egregious of these practices are rare, it should not be assumed that this type of traditional practice is the major source of exposure of the cord stump to invasive pathogens. The large reductions in mortality and omphalitis in the chlorhexidine groups suggest that cord exposure to pathogens is high due to more ubiquitous barriers to achieving hygienic conditions. Examination of the bacteriological profile of the stump of a subset of newborns from the Bangladesh trial (where traditional application to the cord were rarely reported) demonstrated that colonization of the cord with potentially invasive pathogens such as E. Coli, K. Pneumonia, S.aureous, and streptococcus spp is widespread and substantially reduced through chlorhexidine [20].

### Safety of the intervention

Cleansing of the umbilical cord with CHX is considered safe [21,22]. CHX is a broad spectrum antiseptic extensively used in dental, obstetric and surgical scrubs. It has also been used in obstetrics, peri-partum, perineal and vaginal washes in concentrations as high as 4% [23]. CHX is currently included in WHO’s Essential Drugs List and is the antiseptic of choice for cord care in hospitals [17]. Despite widespread use in clinical and community settings for over 30 years, no significant adverse events associated with topical applications to the cord stump have been reported in neonates [22]. No side effects were reported in any of the included studies in this review.

### Implications for policy

Infections contribute to about one third of 3 million annual neonatal deaths in developing countries [18]. The risk of infection is significantly high in the case of home deliveries, often attended by unskilled traditional birth attendants with unclean delivery practices [24] and potentially harmful family practices for cord care [4]. The widespread application of harmful substances to the cord stump, seen in many poor urban and rural settings, can facilitate the entrance of microorganisms and skin flora into blood stream leading to infection and omphalitis [5]. Compounding these problems are high rates of low-birth-weight and preterm birth, often associated with increased risk of infections [25]. This review makes an important contribution by identifying an intervention for appropriate umbilical cord care in community settings in developing countries, and suggests that application of 4% CHX to newborn umbilical cord can substantially reduce cord infection and neonatal mortality. According to CHERG rules, an estimate for all-cause mortality was used for inclusion to LiST model for sepsis mortality. This was due to the fact that there was not enough data for sepsis specific mortality and the overall quality of data was “low”. Based on CHERG method paper, estimate for all-cause mortality (i.e. 23 % reduction) was used by downgrading overall quality assessment from “moderate” to “Low”. We believe that this simple and inexpensive intervention, if applied at scale, has the potential to significantly reduce neonatal mortality in developing countries.

### Conclusions and implications for future research

Application of 4% CHX to umbilical cord of newborn leads to a reduction of 23% (95% CI: 6 % to 37%, random effect model) in all-cause neonatal mortality compared no CHX. Application of CHX leads to reduction in omphalitis in a range of 27% to 56% compared to controls depending on severity of infection. The most prominent protective effects of CHX occur in first week of life. The impact decreases after first week of life, but remains significant throughout neonatal period.

All three included studies were conducted in South Asia and one included a delivery strategy using birth kits and usage by family members. Further studies are being conducted in community settings in Africa to assess the intervention and potentially replicate findings [14,15]. Once available, those data should also inform the global evidence-base. Future studies should also evaluate delivery methods of CHX in large community programs. Nevertheless, this meta-analysis provides promising information of relevance to policies for newborn care in South Asia, especially among home births and high-risk situations and calls into question the current WHO recommendations for dry cord care in all settings. Finally, the protective benefit of chlorhexidine appeared to be similar among facility-born and home-born babies. This finding is not surprising given the challenges of achieving hygienic practices in facilities during labor, delivery and the immediate postpartum period, and the fact that in the vast majority of cases facility-born babies are very quickly discharged into the same conditions as home-born babies.

## Competing interests

All authors declare that they do not have any conflict of interest

## Contribution of authors

ZAB put forward the original idea for the meta-analysis and supervised the analyses and manuscript writing. AI did the literature search. A I and L M conducted the analysis and wrote the manuscript. AHB, SEA, JMT^2^, SKK, RS, SC and REB helped in analyses and revision of manuscript.

## Supplementary Material

Additional file 1 Risk of bias in included studies Click here for file

## Declarations

The publication costs for this supplement were funded by a grant from the Bill & Melinda Gates Foundation to the US Fund for UNICEF (grant 43386 to "Promote evidence-based decision making in designing maternal, neonatal, and child health interventions in low- and middle-income countries”). The Supplement Editor is the principal investigator and lead in the development of the Lives Saved Tool (LiST), supported by grant 43386. He declares that he has no competing interests.

This article has been published as part of *BMC Public Health* Volume 13 Supplement 3, 2013: The Lives Saved Tool in 2013: new capabilities and applications. The full contents of the supplement are available online at http://www.biomedcentral.com/bmcpublichealth/supplements/13/S3.
