# Supplementary material for: The effect of umbilical cord cleansing with chlorhexidine on omphalitis and neonatal mortality in community settings in developing countries: a meta-analysis
Source: BMC Public Health. 2013 Sep 17;13(Suppl 3):S15. doi: 10.1186/1471-2458-13-S3-S15 (PMC3847355; doi:10.1186/1471-2458-13-S3-S15)
Supplement: Additional file 1 — Risk of bias in included studies [file 1471-2458-13-S3-S15-S1.pdf]

Web Table 1: Risk of bias in included studies

Parameter	Nepal trial	Bangladesh trial	Pakistan trial
**Sequence generation**	**Low risk**<br>**Quote:** “Sectors (clusters) were randomised with a computerized random number generator, with assignment blocked on tertiles of sector-wise infant mortality risk estimated with data from a previous NNIPS study.”	**Low Risk**<br>**Quote:** “Randomization was done by generating random allocation sequences in STATA (version 10 9.2)”	**Low risk**<br>**Quote:** “Using a 2x2 factorial design, the clusters were randomly allocated to one of the four groups using computer-generated random numbers”
**Allocation concealment**	**Low Risk**<br>**Comment:** Since it is a cluster randomized trial, allocation concealment should not be an issue as in this design all clusters are randomized at once.	**Low Risk**<br>**Comment:** Since it is a cluster randomized trial, allocation concealment should not be an issue as in this design all clusters are randomized at once.	**Low Risk**<br>**Comment:** Since it is a cluster randomized trial, allocation concealment should not be an issue as in this design all clusters are randomized at once.
**Blinding**	**Low risk**<br>**Quote:** “Study investigators, analysts, project field workers, and participants were masked to the chlorhexidine and	**High Risk**<br>**Quote:** “Workers and participants in the study were not masked to group allocation.”	**High Risk:**<br>**Quote:** “The CHWs maintained close liaison with TBAs, were informed of all pregnancies 22 and

**Parameter**	**Nepal trial**	**Bangladesh trial**	**Pakistan trial**
	to the soap and water treatments.”		provided TBAs with birth kits as per requirement” Comment: Most probably not done
**Missing data**	**Low risk** **Comment:** Number and reasons for those excluded and loss to follow up were given in the text.	**Low risk** **Comment:** Number and reasons for those excluded and loss to follow up were given in the text.	**Low risk** **Comment:** Number and reasons for those excluded and loss to follow up were given in the text.
**Selective outcome reporting**	**Low risk** **Comment:** All the *a priori* mentioned outcomes in protocol were reported.	**Low risk** **Comment:** All the *a priori* mentioned outcomes in protocol were reported.	**Low risk** **Comment:** All the *a priori* mentioned outcomes in protocol were reported.
